# Health inequalities under decentralized governance: challenges in resource allocation and funding in Greece

**DOI:** 10.3389/frhs.2025.1701887

**Published:** 2025-11-14

**Authors:** Stefanos Karakolias, Nikolaos Polyzos

**Affiliations:** 1 Department of Nursing, Democritus University of Thrace, Alexandroupolis, Greece; 2 Department of Medicine, Democritus University of Thrace, Alexandroupolis, Greece

**Keywords:** decentralization, regional health authorities, primary healthcare, resource allocation, funding, health inequalities

## Abstract

**Background:**

Decentralization in health systems enhances responsiveness and equity but is often accompanied by uneven implementation and resource disparities. Greece' health system has undergone successive phases of decentralization, culminating in a transformation in 2015 when regional health authorities (RHAs) assumed operational responsibility for public primary healthcare (PHC). This study presents the first comprehensive assessment of this transition, examining funding adequacy and resource allocation across RHAs.

**Methods:**

Financial and operational analyses were performed to assess disparities among RHAs and between RHAs and hospitals. Data were drawn from publicly available sources, including financial statements, reports from the Ministry of Health, and national statistics. The analysis examined patient visits, staffing levels, infrastructure, funding, labor productivity, and efficiency across health regions.

**Results:**

Between 2018 and 2023, patient visits declined at most RHAs. Staffing composition shifted toward nursing personnel, while medical staff numbers declined. Substantial intraregional and interregional disparities were observed in service utilization, staffing, infrastructure, funding, labor productivity, and efficiency. Hospitals continued to absorb a large share of PHC demand and funding, whereas RHA units held markedly fewer assets and received lower financial support. Funding imbalances among RHAs were evident, and the overall negative return on assets indicated systemic underfunding of public PHC.

**Conclusion:**

The ongoing decentralization of Greece's health system faces structural challenges, including overlapping territorial jurisdictions and uneven, occasionally insufficient, resource allocation. These challenges hinder progress toward health equity. Policy interventions should prioritize evidence-based resource allocation, standardized financing frameworks, and strengthened PHC integration to promote equitable and sustainable healthcare delivery under decentralized governance.

## 1 Introduction

Decentralization in health systems is the process of transferring responsibilities and decision-making authority from the central to the local levels. It has been extensively implemented across the world to improve health system responsiveness, performance, and health outcomes (1, 2). The process typically encompasses political, administrative, and fiscal dimensions, with varying degrees of autonomy granted to local authorities (1).

Political decentralization transfers policymaking authority to local governments and elected representatives, enhancing democratic governance by aligning health policies with community needs (3). It assumes citizen and stakeholder participation in local planning to address health constraints, opportunities, and best practices (4). Administrative decentralization distributes management responsibilities across government levels, transferring decision-making over health service delivery to local agencies or facilities. It grants these entities the autonomy to manage resources, implement programs, and oversee operations (5). Fiscal decentralization involves distributing financial resources and authority over revenue generation and spending to local governments. This ensures that local governments have the means to manage health services effectively and can enhance governance by reducing corruption and increasing accountability (6).

Research on the effects of decentralization on health systems and public health has yielded mixed results (7). Some studies indicate positive impacts on population health outcomes (8), health security capacity, and user satisfaction (9), as well as improved responsiveness during health crises (10). In contrast, other studies suggest that decentralization may exacerbate regional disparities and inequities (11). In general, the effectiveness of health system decentralization appears to be context-dependent and influenced by factors such as local governance capacity, resource availability, and implementation processes (2, 12).

### 1.1 Challenges when decentralizing

The first major challenge throughout the decentralization process is resource allocation, including of personnel, infrastructure, and financial resources (13). Equitable resource allocation refers to the fair and balanced distribution of these resources across regions and health system levels to ensure equal access to quality healthcare for all populations. A couple of studies from China revealed disparities between urban and rural areas, as well as between hospitals and primary care centers, with the former occupying relatively more beds, nurses, and equipment in both cases (14, 15). Similarly, a study from Indonesia showed that provinces most favored in terms of resource allocation provide superior services and produce better health outcomes (16). As such, efforts made toward decentralization without equitable resource allocation have proven to be fruitless. This occurred even in the case of experienced and strongly decentralized systems, with Switzerland being a representative example (17). Interestingly, a study from Spain found that the lasting benefits of decentralization accrued only to regions with full fiscal and political power, which were also among the wealthiest (18).

As implied previously, the ability of local health authorities to provide adequate health services strongly depends on their financial capacity (19). For example, in Italy, “linear” cuts due to the global economic crisis in the 2010s led to approximately one-third of regional governments—mainly in the central and southern parts of the country—facing large financial deficits (20). Similarly, many local public health authorities in Canada have reported insufficient funding for adaptation activities, particularly in relation to climate change (21). Limited financial resources hinder the implementation of approved programs and often compel public managers to purchase services from the private sector at higher prices (22), a contradiction in itself under conditions of budget scarcity. Consequently, underfunding is the second major challenge in the decentralization process.

Several factors contribute to these challenges. First, inequities in resource allocation often stem from systemic biases that favor certain regions due to historical and socioeconomic factors (23). Second, disparities in the technical expertise and institutional robustness of local authorities frequently prompt heterogeneous implementation of nationally mandated policies (24). Addressing these issues requires equitable policies that correct systemic imbalances and promote inclusive distribution. Technological solutions, such as learning-based allocation systems, alongside governance reforms, can enhance efficiency and reduce dependence on centralized data (24). Furthermore, modernized data systems support sustainable investment flows (25). Integrating these strategies demands a multidisciplinary approach and a strong commitment to equity and sustainability.

### 1.2 Decentralization in the Greek health system

Greece's National Health System (ESY) has been undergoing continuous reform efforts, including attempts at decentralization, to address ongoing challenges and crises. Even before its establishment in 1983, the local health system experienced decentralization, which was recognized as the first phase of decentralization (26). The second phase lasted from 1983 to 2001, while the third phase spans the past three decades (26). In 2000, the Greek government announced numerous reforms aimed at decentralizing the ESY, creating a unified financing system and reorganizing hospital management and primary care (27). In 2001, 17 Regional Health Systems (PeSY) were created. Two years later, the centers were renamed Regional Health and Welfare Systems (PeSYP), and another two years later, they were yet again renamed Regional Health Directorates (DYPe) (26). In 2007, the 17 DYPe were merged into seven Health Regions (YPe). This decentralization structure remains effective to date. Hereafter, in this study, the seven YPe will be referred to as regional health authorities (RHAs).

While surprisingly few studies have assessed the decentralization of the ESY, those that have consistently converge on moderate levels of decentralization, since RHAs exercise limited power (28–30). Their role is principally advisory and supervisory (28), although they are intended to carry out extensive healthcare planning and organization. Moreover, their structure is still considered unclear (31), as it is inconsistent with administrative regions, which means that administratively unified areas (e.g., regional units and municipalities) are split between different RHAs. Athanasiadis et al. (29) were exceedingly insightful when they captured the views of former directors of RHAs on the obstacles to effective health decentralization. The following are some of the mentioned obstacles: 1) limited transfer of power, especially in terms of political and fiscal autonomy; 2) lack of political support, since a mentality of centralized control prevails; 3) bureaucratic challenges, mainly referred to as overregulation, such as sharing responsibilities with the Ministry of Health (MoH), leading to delays in decision-making; 4) discontinuity in health policies as a result of frequent changes in governments, ministers, and directors of RHAs; 5) financial burdens as reflected by dependent budgets and the absence of budgetary control; 6) the short tenure of directors; and 7) resistance from various stakeholders (e.g., civil servants, trade unions, and local political figures).

The year 2015 was a milestone year for RHAs in Greece, when they absorbed public primary care units that were previously included in the organizational charts of hospitals. Since then, RHAs have continued to supervise hospitals and provide primary care within their catchment areas. This type of care is delivered through urban and rural health centers along with their satellite units, namely, regional medical offices and local health units (TOMY) (hereafter referred to as RHA units). RHAs operate as legal entities of public law (NPDD), similar to hospitals; thus, they have their own budgets and staff, and publish financial statements annually. Therefore, they enjoy increased administrative freedom and accountability. In this context, the new era starting in 2015 may be recognized as the fourth phase of decentralization.

Although this phase marks a notable advancement in decentralization, its outcomes remain insufficiently understood. Accordingly, this study evaluates this most recent decentralization effort, focusing specifically on whether RHAs have received adequate funding and equitable resource allocation. Examining these factors is critical for understanding whether the current decentralization model is associated with improvements in health system responsiveness and reductions in regional inequalities. By analyzing financial and operational data across RHAs, this study seeks to identify persistent structural challenges that may be undermining the intended benefits of decentralization and perpetuating health inequities across different regions of Greece.

## 2 Methods

### 2.1 Study design and data collection

This study employed a mixed methods approach combining financial and operational analyses to evaluate the decentralization of the ESY, with a focus on funding adequacy and resource allocation for RHAs. In fact, this study aimed to highlight geographical differences both among RHAs and between RHAs and hospitals. Hospitals were included because they complement RHA units by offering outpatient care through regular, evening, and emergency clinics, potentially creating a substitution effect within public primary healthcare (PHC).

The first step involved data collection to determine the appropriate financial and operational indicators. From the outset, it became apparent that the data were fragmented and incomplete. Consequently, data from multiple sources were consolidated, and the most recent and comparable data of each source were utilized.

Financial data were obtained from the 2022 published financial statements of RHAs and public hospitals via the Transparency Portal (32), establishing a static financial framework. This approach was adopted primarily because 2022 provides the most recent fiscal year with complete data for both RHAs and hospitals. Fiscal years 2020–2021 were excluded to control for the confounding effects of the COVID-19 pandemic, while fiscal years 2015–2017 were omitted due to significant volatility during the gradual transfer of assets from hospitals to RHA balance sheets.

Operational data included patient visits, staffing, and catchment population. Information on visits to RHA units and hospitals between 2018 and 2023 was obtained from the Greek Ministry of Health (33). Staffing data for RHA units (2021–2023) were drawn from the Hellenic Statistical Authority (ELSTAT) (34), and population figures were based on the 2021 national census conducted by ELSTAT (35).

### 2.2 Data analysis and indicators

The analysis began with longitudinal data assessing operational trends in Greece's PHC across two levels. The first level examined outpatient visits to public PHC units, encompassing RHA units and designated hospital departments. The second level focused on staffing within RHA units, categorized into medical, nursing, and other personnel.

Subsequently, the analysis examined potential substitution effects within public PHC by assessing the 2022 share of RHAs in key operational and financial measures, including patient visits (disaggregated into emergency and routine), assets (fixed and current), balance sheet equity, and state subsidies.

Finally, the analysis focused on 2022 financial and operational indicators to elucidate geographical variations among RHAs. Data availability and the study's objectives guided the selection of indicators, as detailed in Supplementary Table S1. These indicators examine staff and infrastructure allocation, funding, labor productivity, and efficiency.

### 2.3 Data adjustments and limitations

Population data were adjusted to align administrative divisions with health regions. This adjustment was required because population statistics in Greece are reported according to the country's 13 administrative regions, which are, by design, smaller than the 7 health regions. Algorithmic adjustment was unnecessary, as administrative divisions are typically nested within specific RHAs, ensuring clear and non-overlapping boundaries. The main exception concerned the third and fourth RHAs, both operating within the Thessaloniki regional unit. The primary challenge involved allocating the approximately 1.1 million inhabitants of this area, which includes the city of Thessaloniki, Greece's co-capital. Given that both RHAs oversee roughly half of the city's public healthcare units, it was assumed that the total catchment population of the Thessaloniki regional unit was evenly divided between the third and fourth RHAs.

No missing, inconsistent, or non-comparable data were identified across the sources used in this study, ensuring a coherent dataset for analysis. The only exception was the second RHA, for which financial statements were available only up to 2018; consequently, this region was excluded from calculations of financial indicators and national averages. Overall, the completeness and consistency of the data support the reliability of the quantitative comparisons and findings.

### 2.4 Ethical considerations

This study utilized publicly available data and did not involve human subject research, thereby not requiring ethical approval.

### 2.5 Statistical analysis

Descriptive statistics were employed to examine temporal trends and cross-sectional regional variations, using Microsoft Excel. The application of inferential statistical techniques was precluded by the aggregate nature of the data. For instance, mean values for indicators such as “visits per doctor” were computed from total regional figures by dividing the total number of visits by the total number of doctors within each RHA, rather than by averaging individual-level observations.

## 3 Results

### 3.1 Trends in service utilization and staffing in primary care

**Figure 1:**
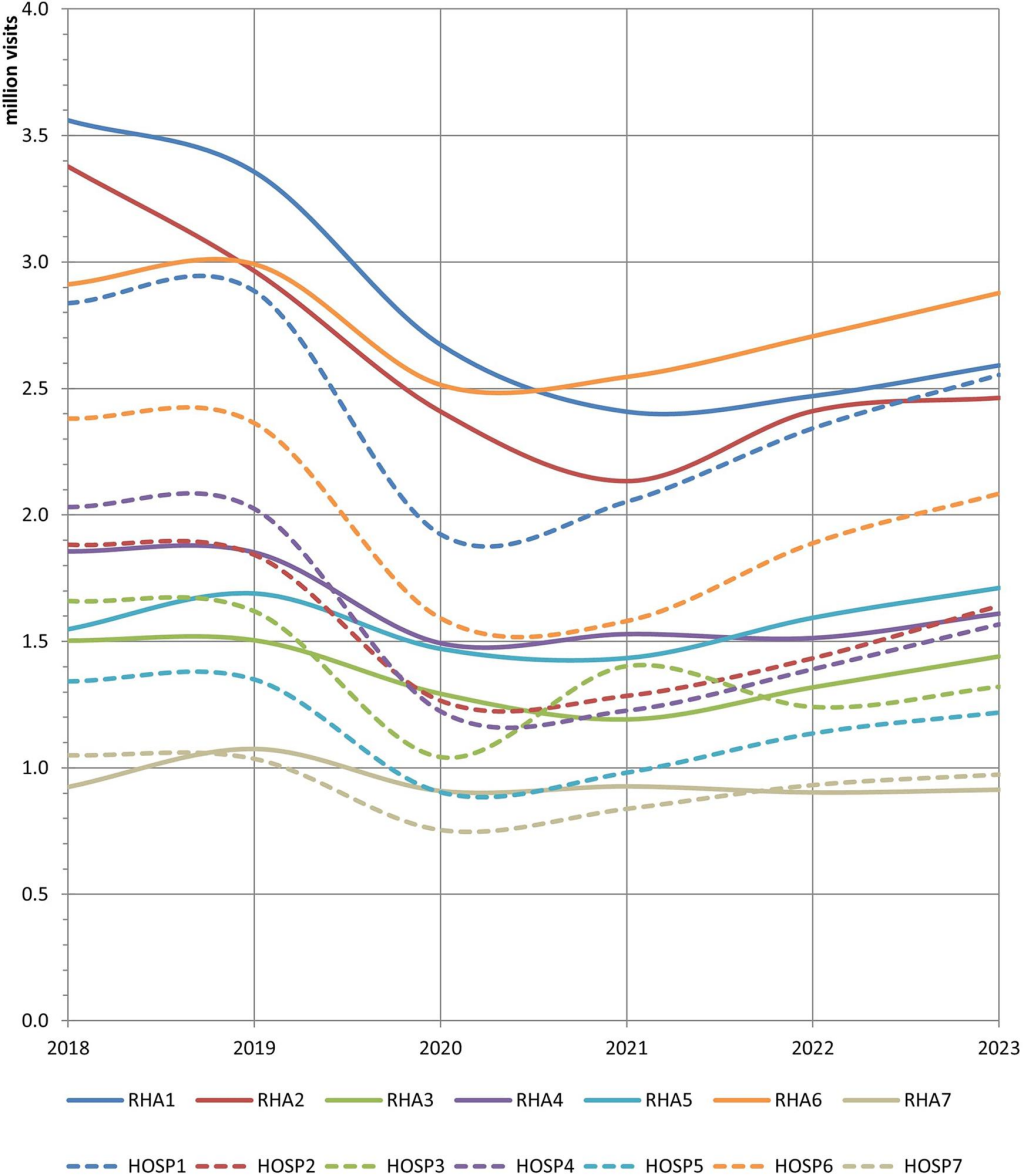
Outpatient visits to RHA units and hospitals, 2018–2023.

Figure 1 presents the output of public PHC, as reflected by the number of outpatient visits to RHA units and hospitals between 2018 and 2023. At first glance, the total number of RHA visits reached 13.6 million in 2023, declining by 13.2% from 2018 to 2023. The number of hospital visits was 11.4 million in 2023, which also indicated a decline (−13.8%). The COVID-19 pandemic had a significant negative impact on both aspects; however, a relatively moderate rebound occurred thereafter. This downward trend profoundly affected the first and second RHAs, as the decline in the number of RHA visits exceeded 27% in both cases. Notably, in the first health region, the gap between the RHA and hospital visits was almost closed by 2023. In contrast, the fifth RHA was the only one that managed to limit the effect of the pandemic and ended the examined period with 10.6% more visits than before. The same figure reveals three distinct groups of RHAs with respect to service production in the most recent year: Each RHA in the first group (1st, 2nd, and 6th) received approximately 2.5–2.9 million visits annually; RHAs 3–5 formed the second group with 1.4–1.7 million visits each, while the seventh RHA was substantially the smallest.

**Figure 2:**
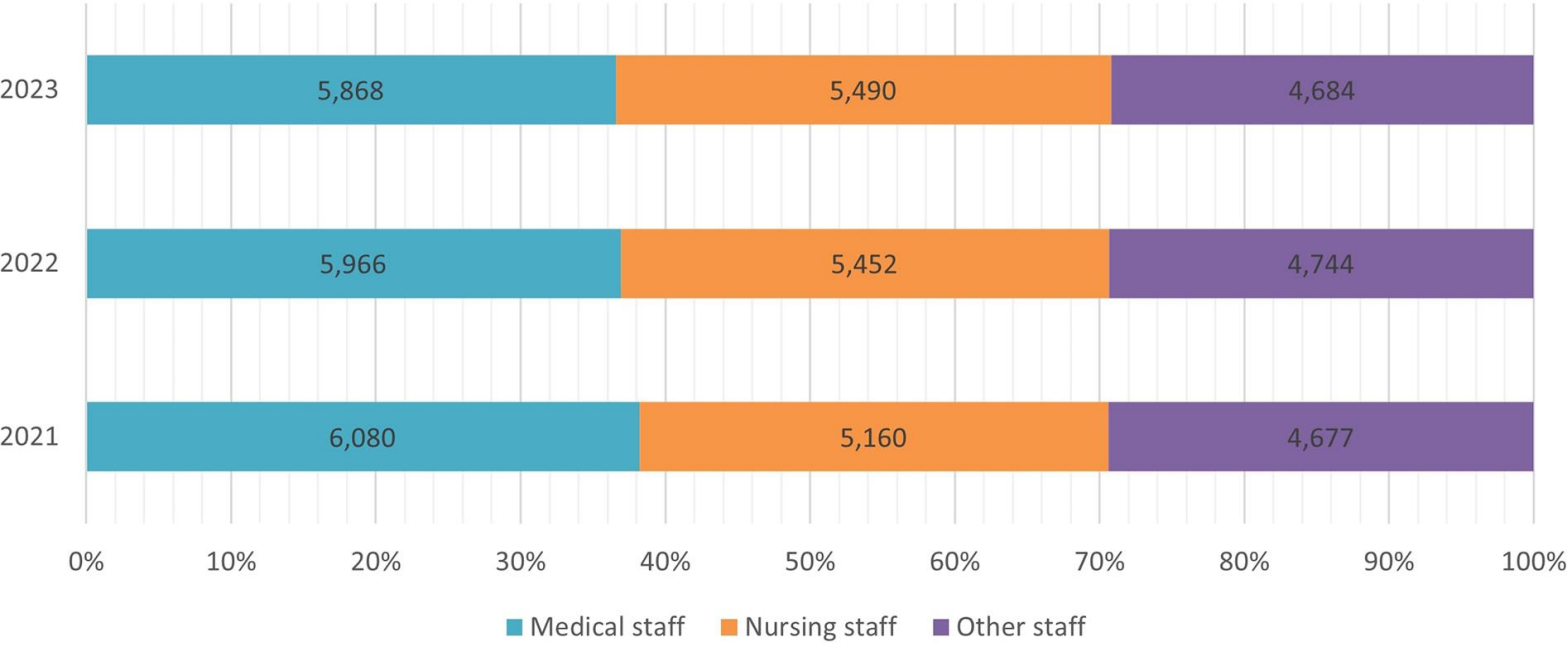
Staff employed at RHA units, 2021–2023.

Figure 2 illustrates how employment in the RHA units changed between 2021 and 2023. The total number of staff amounted to 16,042 employees in 2023, an increase of 0.8% compared to 2021. The number of other (non-medical) staff members remained stable during this period. However, the evolution of medical staff was diametrically opposed to that of nursing staff. The former declined by 3.5% (212 fewer doctors), whereas the latter increased by 6.4% (330 more nurses). Thus, the composition of manpower in public PHC changed in favor of nurses, who are about to outnumber doctors in the near future. The most recent (2023) composition included 37% medical, 34% nursing, and 29% other staff members.

### 3.2 Intraregional and interregional disparities between hospitals and RHA units

Table 1 shows the key operational and financial figures of RHAs for 2022 as a percentage of the total, which is the sum of RHA units and public hospitals. The preliminary finding is that 55.5% of both regular and emergency (i.e., total) visits were made to RHA units. These providers appeared to receive more visits for routine care (61.9% of the total) but fewer visits for emergency care (41.4% of the total). However, substitution within public PHC varies significantly among health regions. For example, in the third and seventh regions, hospitals received nearly 7 out of 10 emergency visits, whereas in the first and second regions, they received nearly half of them. Similarly, hospitals located in the second and sixth RHAs received only 3 out of 10 regular visits, but their counterparts in the first RHA received almost half of that. In short, public hospitals appear to provide a substantial portion of public PHC; therefore, they are expected to absorb a substantial portion of public financial resources for PHC. This is confirmed by the results presented in Table 1. In terms of the 2022 book value, the fixed assets of RHAs represent a small share of the total fixed assets installed in both RHAs and public hospitals, in most cases below or near 5%. This indicates that RHA units lack critical infrastructure, including premises, medical equipment, furniture, and vehicles, compared to hospitals. Notably, after removing the sixth RHA, which has a high-value agricultural property, all the fixed assets of RHAs are barely half as valuable as those of the average hospital in their catchment area. In other words, the average 90 PHC facilities per health region possess fixed assets comparable in value to those of a small to medium-sized hospital in their respective jurisdiction.

**Table 1:** Operational and financial figures of RHAs as shares of total figures, 2022.

Indicator	RHA							Total
	1	2	3	4	5	6	7	
Total visits to RHA units as % of total visits	51.3	62.7	51.5	52.1	58.4	58.9	49.2	55.5
Emergency visits to RHA units as % of total visits	48.3	48.2	27.7	42.7	39.5	38.6	32.6	41.4
Routine/regular visits to RHA units as % of total visits	52.9	69.0	61.3	57.2	65.9	67.8	56.1	61.9
RHA fixed assets as % of total fixed assets	5.4	n/a	4.2	3.6	6.2	12.2	3.3	n/a
RHA total assets as % of total assets	7.6	n/a	2.3	3.0	3.9	6.4	2.0	n/a
RHA equity as % of total equity	7.6	n/a	2.1	2.6	4.1	6.4	2.0	n/a
RHA other revenue as % of total other revenue	22.8	n/a	16.1	16.2	25.8	25.4	21.4	n/a

Table 1 reveals a similar situation for total assets, which means that not only the vast majority of fixed assets but also current assets, such as medical inventories and cash, are concentrated in public hospitals. The corresponding result for equity is almost identical to that for total assets. The fact that equity is formed by state subsidies for investments and retained earnings, the latter of which incorporate state subsidies for salaries and operational expenses, reinforces the finding that RHAs are underfunded compared to public hospitals. This is not absolute with respect to state subsidies for salaries and operational expenses, as the account entitled “other revenue,” which mirrors this kind of subsidy, is allocated more generously to RHAs. For instance, the fifth and sixth RHAs achieved more than one-fourth of the total other revenue. Furthermore, the financial figures in Table 1 provide evidence of financing inequalities among RHAs, especially in the case of the third and fourth RHAs.

### 3.3 Interregional disparities among RHAs

The inequalities among RHAs become more apparent in the results shown in Table 2. Initially, significant discrepancies were observed among the health regions in terms of staff allocation. More precisely, RHAs employ 5.7 doctors/10,000 population and 5.2 nurses/10,000 population, on average. However, the second RHA, which coordinates the southern regions of Attica and the islands of the Aegean, appears to lack both medical and nursing staff members. The shortage of nurses in this area is remarkable, as their density (3.3 nurses/10,000 population) is half or less than half of that in other three health regions. Conversely, the sixth RHA, which covers the largest geographical area, employs the highest proportion of doctors (7.5/10,000 population) and nurses (6.8/10,000 population). On average, approximately 15 employees are appointed per 10,000 population; however, the two aforementioned RHAs deviate by ±4 employees.

**Table 2:** Operational and financial indicators per RHA, 2022.

Category	Indicator	RHA							Total
		1	2	3	4	5	6	7	
Staff allocation	Doctors per 10,000 population	5.7	4.3	4.8	5.5	6.2	7.5	6.8	5.7
	Nurses per 10,000 population	4.0	3.3	5.7	6.6	6.7	6.8	4.3	5.2
	Other staff per 10,000 population	4.3	4.0	4.4	3.8	4.8	5.3	6.1	4.5
	Total staff per 10,000 population	14.0	11.6	14.9	16.0	17.7	19.6	17.3	15.4
Infrastructure allocation	Fixed assets per capita (€)	14.8	n/a	6.4	6.1	14.6	31.5	5.4	14.8
	Total assets per capita (€)	94.9	n/a	33.6	40.9	44.0	73.0	36.5	60.5
Funding	Equity per capita (€)	82.1	n/a	27.2	30.8	41.2	63.4	32.7	51.9
	Other revenue per capita (€)	42.3	n/a	50.3	52.1	60.5	67.7	69.8	55.1
	Equity per employee (€)	58,484.8	n/a	18,209.2	19,250.5	23,342.8	32,324.6	18,957.5	31,569.1
	Other revenue per employee (€)	30,123.9	n/a	33,742.6	32,545.1	34,236.2	34,476.8	40,413.1	33,534.0
Productivity	Visits per doctor	2,047.8	2,527.4	2,329.3	1,904.0	2,162.5	2,111.2	2,120.2	2,165.0
	Visits per nurse	2,888.5	3,262.7	1,970.7	1,590.0	1,994.7	2,317.3	3,345.3	2,369.1
Efficiency	Revenue per visit (€)	6.6	n/a	3.2	4.3	2.3	3.2	1.2	6.6
	Revenue per physician (€)	13,582.9	n/a	7,348.5	8,106.8	5,035.1	6,661.4	2,496.9	8,040.6
	Revenue per employee (€)	5,497.0	n/a	2,367.3	2,805.8	1,756.2	2,541.6	986.7	2,966.5
	Production cost per visit (€)	44.1	n/a	42.5	52.2	42.8	40.5	41.1	43.7
	Total cost per visit (€)	47.5	n/a	48.3	54.4	44.3	46.3	47.7	47.8
	EBIT/total assets (ROA)	−5.5%	n/a	−0.6%	−1.9%	10.3%	−0.8%	6.9%	−1.3%

According to Table 2, similar discrepancies apply to infrastructure allocation. As mentioned earlier, the sixth RHA has a high-value agricultural property; thus, the values of fixed and total assets per capita are inflated compared to other RHAs. However, in the Athens area (first RHA), each resident also enjoys assets of higher value (€94.9 per capita); hence, there is evidence of centralization in terms of assets in this area. In addition, Table 2 shows the differences in funding. The seventh RHA emerges as the most favored with respect to state subsidies intended for staff payroll and operational expenses, as its “other revenue” is proportionally higher, regardless of whether it is expressed as a ratio of the catchment population or staff. Accordingly, the first RHA appears to prevail in terms of total subsidies, as its unit equity far outnumbers that of the others. Evidently, the increased funding in this area was used to acquire either more assets or more valuable assets, as suggested by the infrastructure allocation.

As shown in Table 2, labor productivity is another field of imbalance among health regions. The second RHA, with the aforementioned staffing constraints, stood out in terms of the annual number of visits per doctor and nurse (2,527 and 3,263, respectively). Similarly, the third RHA was distinguished for productivity of doctors (2,329 visits) and the seventh RHA for productivity of nurses (3,345 visits). The fourth RHA, in contrast, had the lowest productivity values, deviating significantly from the average. This was not due to higher staffing, but rather a substitution effect, as only 52.1% of total visits were made to RHA units, shifting roughly 100,000 appointments annually to hospitals.

Moreover, Table 2 confirms that the first RHA not only receives relatively more subsidies (see funding indicators) but also achieves much higher regular revenue than other RHAs (6.6 €/visit). This cannot be attributed to higher productivity but rather to a more complex case mix that leads to higher income from social insurance. In terms of cost management, the average production cost was nearly 44 €/visit, but most RHAs converged at slightly higher than 40 €/visit. The total cost (i.e., production cost plus administrative expenses) ranged between 47 and 48 €/visit in most cases. The fourth RHA was an exemption, as both cost measures exceeded 50 €/visit. Moreover, the fact that the cost per case was not lower for the RHAs with the most visits (6th, 1st, and 2nd RHA) contradicts the economies-of-scale assumption.

The last indicator in Table 2, also referred to as return on assets (ROA), reflects efficiency in terms of how much earnings before interest and taxes (EBIT) are generated per monetary unit of assets. Once this ratio became negative, it implied that expenses surpassed total revenue (regular revenue plus state subsidies); therefore, the corresponding RHA was underfunded. This applies to the first, third, fourth, and sixth RHAs. Nonetheless, the fifth and seventh RHAs were particularly efficient, although the efficiency was not associated with exceptional productivity or higher unit revenue. Thus, overfunding through subsidies is evident in these regions. In short, the negative mean value for ROA (−1.3%) indicates the insufficient financing of RHAs in general.

## 4 Discussion

The pursuit of health equity, which involves the fair and just distribution of resources and opportunities for health, is a central goal of health policy. Decentralization is frequently advocated as a mechanism to advance this goal by enhancing the responsiveness of health systems to local needs. This study examined the ongoing phase of decentralization within the ESY, initiated in 2015 when RHAs assumed direct ownership and management of public PHC units. This reform was envisioned as a means to mitigate regional disparities. However, the findings of this study are consistent with prior research indicating that the effectiveness of decentralization is highly context-dependent and may, paradoxically, exacerbate inequalities if underlying structural deficiencies remain unaddressed.

A prominent structural issue identified is the conflicting and illogical territorial division of RHAs, placing serious restrictions on the coordination and development of integrated care (28, 29, 31). Northern Greece has emerged as a representative case in which the third and fourth RHAs have overlapping responsibilities across the regional unit of Thessaloniki, where more than 10% of the country's population resides. Moreover, both RHAs have their headquarters in Thessaloniki; therefore, equality in coordination across their catchment areas is questionable. For example, the distance between the headquarters of the fourth RHA and its most remote health center is 450 km. The same applies to the sixth RHA, which covers practically the entire western part of Greece (i.e., more than 700 km in a straight line). Inequalities between health regions may also refer to the catchment populations. For instance, each of the first two RHAs in Greece covers more than 2 million people, and both cover more than 40% of Greece's total population. Thus, even in terms of service production, Greece has three large, three moderate, and one small RHA. Consequently, policymakers should evaluate the boundaries and/or number of health regions more frequently.

Inequalities among health regions may extend beyond service production, catchment populations, and geographical areas. International experience features unequal resource allocation as a prevalent issue. This study documented one unusually understaffed RHA (second), another relatively overstaffed (sixth), two RHAs with materially more valuable assets (first and sixth), and two RHAs with relatively ample financing through state subsidies (first and seventh). Moreover, the extent to which RHAs deviate from each other with respect to resources reveals that there are no specific benchmarks or patterns for their equitable distribution. Thus, one could state that resource planning is not a means of ensuring health equity for the Greek population. This assumption cannot be easily rejected, especially considering that the uneven distribution of resources extends to other health services, such as mental health (36) and public hospitals (37). Based on this, the primary policy recommendation is that, rather than relying on the current *ad hoc* distribution, resources should be allocated according to objective criteria and standardized benchmarks that reflect population size, geographical dispersion, and specific local health needs.

Furthermore, this study provides evidence that irrational resource allocation is associated with unequal performance. As such, the utilization of services tends to be higher where staff shortages occur (e.g., in the second health region in Greece). Notably, this increased workload may negatively affect both health professionals and patients (38, 39), especially when considering health systems with a horizontal wage structure and a lack of financial incentives for enhanced accessibility and health outcomes (40). Local market conditions may also affect the labor productivity of the health sector. In the fourth RHA, for instance, patients visit hospital outpatient clinics relatively more often; therefore, the utilization of PHC services of this RHA is lower, which also coincides with a much higher cost per visit in this specific region. At this juncture, there is a need for a more profound integration of PHC structures into the management of emergency cases to enhance efficiency and patient safety. This is an established trend in other health systems (41, 42).

Consequently, the overall efficiency of regional authorities is affected by state subsidies. In Greece, four of the six RHAs recorded losses, while the other two recorded gains in 2022. This reveals regional disparities among health regions in terms of public financing for health. A recent study by Flokou et al. (43) showed that efficiency discrepancies occur even among health units of the same RHA, with TOMY falling short of health centers in terms of technical efficiency. Thus, policymakers should align the efficiency of various health units using relevant indicators.

The macro-level view of our analysis underscores several contemporary developments in Greece's public PHC. First, there was a meager increase in staff employed by RHAs over the period 2021–2023 (0.8%), which embellished the decrease in their medical staff (−3.5%). This is probably associated with the fact that PHC units (under the jurisdiction of RHAs) received 13.2% fewer visits of any kind in 2023 than they did in 2018. The findings of the previous section do not support the idea that patient preferences changed in favor of public hospitals on all occasions, as their outpatient services also declined (−13.8%) over the examined period. Thus, there is evidence that certain groups of patients were redirected to private PHC providers to some extent, as suggested by other researchers (44, 45). This trend undermines the goal of a robust public health system and creates barriers to care for lower-income populations.

Another key finding of this study is that the balance sheets of the RHAs include low-value fixed assets, current assets, and equity, compared with public hospitals. Each RHA resembles a moderate-to-small hospital (of its jurisdiction) in terms of size, despite the fact that the former operates dozens of PHC units. Moreover, the allocation of subsidies, as reflected by the “other revenue” account, reveals that hospitals absorb 75%–85% of them, on average. Thus, there is evidence that state budgets underfund RHAs and, thereby, PHC. In many other low- and middle-income countries, PHC faces financing struggles. This hampers its ability to provide comprehensive, people-centered care; therefore, proposals have been made to increase government spending on PHC (46). However, in the case of budgetary constraints, policymakers should reconsider expanding the sources of financing for PHC, taking advantage of global organizations that fund disease-specific programs (47).

RHAs also appear to be underfunded by social insurance, suggesting a financially unsustainable system that remains heavily reliant on state subsidies, which are often misaligned with actual service needs and regional demands. This is expected, given that RHAs generated an average revenue of only €3.84 per visit in 2022, which is far from the unit cost and common sense. Such insufficient reimbursements have been reported by German general practitioners (48). At this point, another problem emerges, whereby public PHC units are not reimbursed on a cost-per-case basis, such as in the hospital diagnosis-related group (DRG) system. In fact, they are not reimbursed at all in the sense of revenue, as they do not have their own financial management, and resource allocation is based on the centralized budgets of the RHAs. To address this malfunction, researchers have proposed an ambulatory patient groups (APGs) scheme for the Greek public PHC (49).

Certain limitations of this study warrant acknowledgment. First, this study relied on publicly available but fragmented data, as no comprehensive national database exists. This constrained the selection of indicators to those consistently reported across all regions and entities. Second, the analysis was primarily descriptive, focusing on trends and regional variations, since individual-level information on personnel and finances was unavailable, precluding inferential statistical testing. Consequently, while interregional disparities among RHAs and intraregional differences between hospitals and RHA units were identified, differences among specific types of RHA units (e.g., health centers vs. TOMY units) could not be examined. Third, causal inference was not possible due to the aggregate nature of the data; observed relationships between funding adequacy, resource allocation, and regional disparities should therefore be interpreted as associations. Finally, the financial assessment was incomplete at the national level, as financial statements for the second RHA were available only through 2018.

## 5 Conclusion

This study provides the first integrated financial and operational assessment of Greece's decentralization bodies (RHAs) since they assumed operational control of public PHC, shedding light on previously opaque dimensions such as production inputs, service outputs, and financial performance. Findings indicate that, while decentralization is broadly adopted to enhance local autonomy and responsiveness, enduring structural deficiencies may limit its effectiveness and perpetuate health inequalities. The Greek experience illustrates these challenges, particularly uneven resource allocation and PHC underfunding, which are relevant to other countries implementing decentralized health reforms. A central policy implication is the establishment of a comprehensive monitoring and evaluation framework at the national level, coordinated by the Ministry of Health in collaboration with the RHAs. Such a system should employ composite indices and spatial analyses to identify underserved areas and systematically track disparities in staffing ratios, per capita asset values, subsidies, labor productivity, financial performance, and other metrics beyond those captured in this study that should be regularly measured.

Future research should strengthen these policy efforts by leveraging unit-level data to examine operational and financial variations within health regions, accounting for heterogeneity across service units. In addition, longitudinal or panel studies using rigorous econometric methods can clarify causal links between decentralization, resource allocation, and health inequalities. Investigating how staffing levels and labor productivity influence patient outcomes and workforce performance would provide further insights into strategic planning. Collectively, these lessons highlight that decentralization, when paired with systematic monitoring and evidence-driven resource allocation, can serve as a powerful driver of health equity.

## Data Availability

Publicly available datasets were analyzed in this study. These data can be found here: https://www.statistics.gr/el/statistics/-/publication/SAM03, https://www.statistics.gr/en/statistics/-/publication/SHE06, https://www.moh.gov.gr/articles/bihealth/stoixeia-noshleytikhs-kinhshs, and https://diavgeia.gov.gr/.
