# Supplementary material for: Health inequalities under decentralized governance: challenges in resource allocation and funding in Greece
Source: Front Health Serv. 2025 Nov 14;5:1701887. doi: 10.3389/frhs.2025.1701887 (PMC12660272; doi:10.3389/frhs.2025.1701887)
Supplement: Supplementary file 1 [file Table1.docx]

Supplementary Material

**Supplementary Table 1.** Indicators used to assess geographical variations among RHAs.

| **Category** | **Indicator** | Definition | Formula |
| --- | --- | --- | --- |
| Staff allocation | Doctors per 10,000 population | Number of doctors relative to population served | (Number of doctors / Population) × 10,000 |
|  | Nurses per 10,000 population | Number of nurses relative to population served | (Number of nurses / Population) × 10,000 |
|  | Other staff per 10,000 population | Number of other healthcare staff relative to population served | (Number of other staff / Population) × 10,000 |
|  | Total staff per 10,000 population | Total healthcare staff relative to population served | (Total staff / Population) × 10,000 |
| Infrastructure allocation | Fixed assets per capita (€) | Value of fixed assets per population | Fixed assets / Population |
|  | Total assets per capita (€) | Value of all assets per population | Total assets / Population |
| Funding | Equity per capita (€) | Equity allocated per population | Equity / Population |
|  | Other revenue per capita (€) | Non-equity revenue per population | Other revenue / Population |
|  | Equity per employee (€) | Equity allocated per staff member | Equity / Total staff |
|  | Other revenue per employee (€) | Non-equity revenue per staff member | Other revenue / Total staff |
| Productivity | Visits per doctor | Number of patient visits per doctor | Total visits / Number of doctors |
|  | Visits per nurse | Number of patient visits per nurse | Total visits / Number of nurses |
| Efficiency | Revenue per visit (€) | Revenue generated per patient visit | Total revenue / Total visits |
|  | Revenue per physician (€) | Revenue generated per doctor | Total revenue / Number of doctors |
|  | Revenue per employee (€) | Revenue generated per staff member | Total revenue / Total staff |
|  | Production cost per visit (€) | Cost of delivering one patient visit | Total production cost / Total visits |
|  | Total cost per visit (€) | Total cost (including overhead) per patient visit | Total cost / Total visits |
|  | EBIT/Total assets (ROA) | Return on assets, a measure of profitability | EBIT / Total assets |
